# Preparation and Application of Polymer-Dispersed Liquid Crystal Film with Step-Driven Display Capability

**DOI:** 10.3390/molecules29051109

**Published:** 2024-03-01

**Authors:** Hui Lin, Yuzhen Zhao, Xiangke Jiao, Hong Gao, Zhun Guo, Dong Wang, Yi Luan, Lei Wang

**Affiliations:** 1 Xi’an Key Laboratory of Advanced Photo-Electronics Materials and Energy Conversion Device, School of Electronic Information, Xijing University, Xi’an 710123, China; huilin2021a@163.com (H.L.); zyz19870226@163.com (Y.Z.); 17752920030@163.com (X.J.); guozhun@xijing.edu.cn (Z.G.); 2 Department of Materials Physics and Chemistry, School of Materials Science and Engineering, University of Science and Technology Beijing, Beijing 100083, China; 3 Division of Material Engineering, China Academy of Space Technology, Beijing 100094, China; 4 Key Laboratory of Chemical Additives for China National Light Industry, Shaanxi Key Laboratory of Chemical Additives for Industry, College of Chemistry & Chemical Engineering, Shaanxi University of Science & Technology, Xi’an 710021, China

**Keywords:** polymer-dispersed liquid crystals (PDLC), fluorescent dye, partitioned polymerization, electro-optical properties, step-driven display

## Abstract

The realization of multifunctional advanced displays with better electro-optical properties is especially crucial at present. However, conventional integral full drive-based transparent display is increasingly failing to meet the demands of the day. Herein, partitioned polymerization as a novel preparation method was introduced innovatively into polymer-dispersed liquid crystals (PDLC) for realizing a step-driven display in agreement with fluorescent dye to solve the above drawback. At first, the utilization of fluorescent dye to endow the PDLC film with fluorescent properties resulted in a reduction in the saturation voltage of the PDLC from 39.7 V to 25.5 V and an increase in the contrast ratio from 58.4 to 96.6. Meanwhile, the experimental observations and theoretical considerations have elucidated that variation in microscopic pore size can significantly influence the electro-optical behavior of PDLC. Then, the step-driven PDLC film was fabricated through the exposure of different regions of the LC cell to different UV-light intensities, resulting in stepwise voltage–transmittance (V–T) responses of the PDLC film for the corresponding regions. Consequently, under appropriate driving voltages, the PDLC can realize three different states of total scattering, semi-transparent and total transparent, respectively. In addition, the PDLC film also embodied an outstanding anti-aging property and UV-shielding performance, which makes it fascinating for multifunctional advanced display applications.

## 1. Introduction

Transparent display is being favored as a new generation of display devices with the features of visualizing images on a panel and allowing the observer to see both sides of the view at the same time [1]. In addition, their unique features like having a thin profile, low power consumption, light weight, and so on are enticing [2]. Transparent display in particular has the potential to open up numerous new business dynamics for the ongoing display industry, as well as giving users a wonderful experience of a better quality. Over the last few years, a great deal of research has been conducted on transparent display technology, and a variety of approaches have been developed and put into practice, including plasma display [3], liquid crystal display (LCD) [4], electrowetting display [5], organic light-emitting diodes (OLEDs) [6], cholesteric liquid crystals (ChLCs) [7], and polymer-dispersed liquid crystal (PDLC) [8]. Among these candidates, the latter three are more widely used, yet OLEDs and CHLCs still have some existing drawbacks. In the case of OLEDs, a background of significant ambient light results in reduced visibility due to their self-emitting properties [9]. As well, large sizes are challenging to fabricate, and their reliability is still not good enough. For the ChLCs, the designed driver circuits generally require an elevated reset voltage and a complicated driver mode in that the three states under the action of an electric field are a planar state, a focal conic state, and a homeotropic state. 

Electrically switchable PDLCs are extensively applied in preparation for large-area displays and smart windows equipped with scattering and transparent states in view of their polarization–independence, simplicity of fabrication, and low cost [10,11,12,13,14,15,16,17,18]. PDLCs can be formed using micro- or nano-sized liquid crystal droplets dispersed in a uniform polymer matrix and have emerged as an essential new class of materials for a variety of utilizations in optical devices [19,20,21,22,23]. In general, PDLC presents as a milky-white scattering state on account of the refractive index mismatch existing between the polymer matrix and the liquid crystal (LC) droplets. When applying an electric field to it up to a certain strength, the PDLC film changes from the scattered state to the transparent state, a phenomenon that can be ascribed to the rearrangement of the LC molecules along the direction of the external electric field towards matching the refractive index of each phase within the system [24,25,26,27,28,29]. The reversible switching of electro-optical capability provided PDLC films with various possibilities in fields such as multi-color displays [30], micro-lenses [31], anti-peeping films [32], chemical sensors [33], and organic light-emitting diodes [34]. 

There are four methods to achieve the preparation of PDLC films, which are polymerization-induced phase separation (PIPS), solvent-induced phase separation (SIPS), thermally induced phase separation (TIPS), and the microencapsulation process (MP) [35,36,37]. Of these, PIPS is the most commonly used, as it is relatively simple and fast to produce, requiring only a UV curing process and no other tedious steps. Furthermore, it can control the UV-light intensity to modulate the micro-morphology of the sample to realize differentiated electro-optical properties. Typically, PDLC films tend to require low threshold voltages, fast response times associated with on/off state transition, and high levels of contrast ratios for on/off transmittance to realize the demands of safety in the application process [38,39,40]. As for contemporary PDLC devices, drawbacks such as a high driving voltage, low contrast ratio, and inferior mechanical applicability are still restricting their further deployment and must be carefully considered. Extensive research is being conducted to optimize the properties of these PDLC devices, and various approaches such as regulating polymerization conditions, changing the morphology of the microstructure, and doping functional materials have been utilized to improve their performance [41,42,43,44,45,46,47]. By doping various types of dichroic dyes with different concentrations in the PDLC formulation, Zhao et al. not only realized the adjustment of the morphology, the driving voltage, and the contrast ratio of the PDLC films, but also provided a theoretical basis for obtaining PDLC films with a wider color gamut [48]. Li et al. utilized the method of doping rare-earth nanoparticle GeO_2_, whereby the threshold voltage (V_th_) of PDLC decreased by 36.8% and the contrast ratio increased by 53.7%, which significantly enhanced the electro-optical performance of PDLC film [49]. Ahmad et al. prepared polymer-dispersed liquid crystal (PDLC) films using photo-induced phase separation at a wide range of UV intensities (I = 0.33–1.8 mW/cm^2^) and curing times (t = 120–600 s). The results showed that the increase in UV-light intensity accelerated the phase separation and significantly influenced the final morphology of the LC droplets inside the PDLC. Similarly, enhanced phase separation was observed by extending the curing time [50]. However, with the increasing maturity of the overall whole display technology, there is an urgent demand for the application of more ingenious methods to achieve advanced step-driven displays. 

Conventional PDLC devices have been successful in their application as electronic switching screens for privacy management. However, at present, the integral full drive-based transparent display has been difficult to satisfy the demand for advanced displays, and the existing step-driven technology usually involves complicated alignment processes. Herein, we reported on the preparation and analysis of a novel PDLC film with the capability of producing a step-driven display using the partitioned polymerization strategy. First, the fluorescent material 7-Amino-4-methylcoumarin was introduced into the LC system, which was dedicated to the optimization of the driving voltage and contrast ratio. Thereafter inspired by the fact that light intensity enabled differentiation in the driving voltage, the PDLC device was prepared by exposing the different regions of the LC cell to different UV-light intensities, leading to different voltage–transmittance (V–T) responses of the PDLC device for different regions. Thus, by applying an appropriate driving voltage, three different states, total scattering, semi-transparent, and total transparent, can be realized, respectively. Notably, the novel PDLC device also exhibited brilliant UV-shielding and anti-aging properties, which can empower it to be promising in the advanced display field.

## 2. Results and Discussion

### 2.1. The Effect of Cross-Linker on Electro-Optical Properties of PDLC

**Figure 1:**
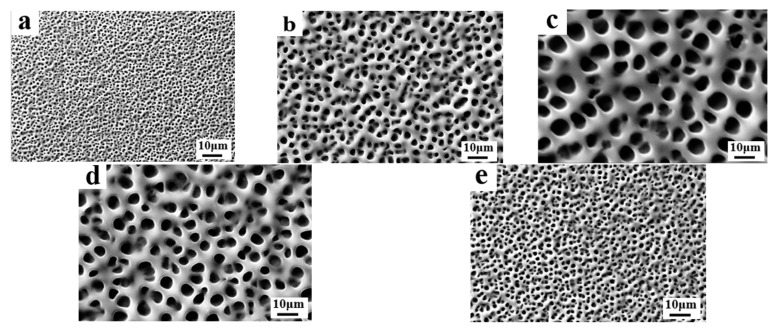
The microscopic mesh distribution of polymer matrix in samples A1–A5: (**a**) 0% IBMA; (**b**) 1% IBMA; (**c**) 2% IBMA; (**d**) 3% IBMA; (**e**) 4% IBMA.

In this part, a commercial PDLC polymer matrix UV-6301 and two monomers, IBMA and TMPTA, were utilized to investigate the effect of different types of cross-linker ratios on the microscopic pore distribution and electro-optical properties of PDLC films. The composition of the samples used for the study is given in Table 1, A1–A5. The IBMA is a monofunctional acrylic monomer featuring a single reactive site, while TMPTA features triple the active sites. By varying the ratio of trifunctional and monofunctional monomers, the crosslinking level of the polymer matrix UV-6301 can be regulated. Figure 1 presents SEM images of the micro-distribution of the polymer meshes in PDLC with different cross-linker ratios. As can be seen from the SEM images, the mesh size gradually increased as the content of IBMA in the system increased from 0% to 2%. However, when the content of IBMA continued to increase to 4%, the mesh size decreased gradually. The existence of this phenomenon can perhaps be attributed to the following reasons: When the IBMA content is low in the system, the diluting effect is predominant, which causes the viscosity of the system to decrease and slows down the phase separation rate, resulting in the gradual increase in microscopic polymer pore size [51]. However, as the content of IBMA reaches a certain value, the further addition of IBMA leads to an increase in the number of cross-linking sites within the system, promoting the formation of a polymer network with a progressively denser pore size. 

**Figure 2:**
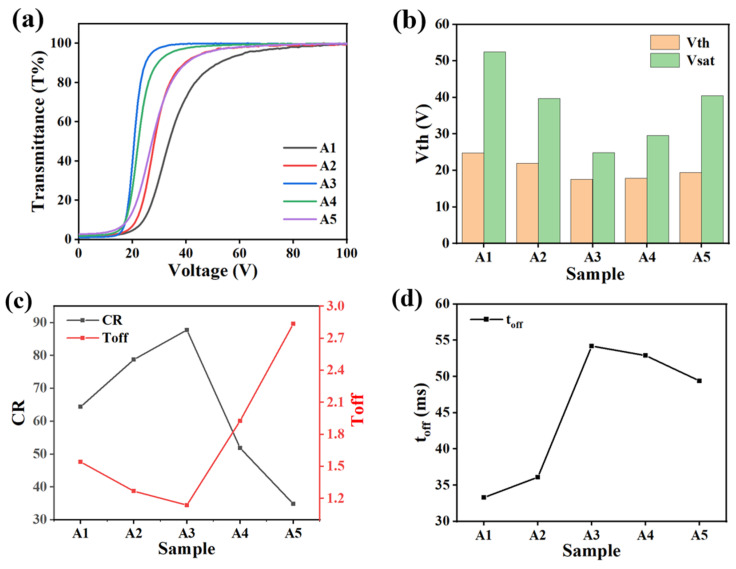
The effect of different cross-linker ratios on electro-optical properties. (**a**) V–T curve; (**b**) V_th_ and V_sat_; (**c**) CR and T_off_; (**d**) t_off_.

Figure 2 illustrates the effect of different crosslinker ratios on the electro-optical properties of PDLC films. Figure 2a shows the voltage–transmittance (V–T) curves for each sample in group A. From the trend of the curves, the variation of the crosslinker ratio did not change the basic electro-optical properties of the PDLC film, and it was still able to achieve the transition from opaque to transparent [51]. As shown in Figure 2b, V_th_ and V_sat_ decreased and then increased with the increase in IBMA content and reached the minimum voltage at 2% IBMA. This phenomenon can be explained using the above analysis of the mesh variation. The size of the aperture was estimated to be negatively proportional to the anchorage force it exerted on the LC molecules. When the content of IBMA varied from 0% to 2%, the pore size of the polymer network gradually became larger and the anchoring force acting on the LC molecules decreased, which resulted in a decrease in the voltage required for the re-orientation of the LC molecules. However, as the IBMA content continued to increase, the polymer pore size gradually decreased and the anchoring force increased, resulting in a higher driving voltage [52]. Figure 2c exhibits the varying curves of the CR and T_off_ for different monomer ratios. As the content of IBMA increased, the T_off_ of the samples first decreased and then increased, reaching a minimum at 2%. Up to this point, the optimal match of the refractive indices between the LC molecules and the polymer matrix was achieved, and the maximum CR was obtained. However, as the content of IBMA was increased further, the refractive indices between the LC molecules and the polymer network were gradually mismatched, leading to an increase in the T_off_ and a decrease in the CR [53]. Figure 2d shows that the t_off_ of samples A1–A5 firstly increased and then decreased with the increase in IBMA content, and the longest t_off_ was observed at 2%, which corresponded to the biggest distribution of the polymer mesh as well.

### 2.2. The Effect of LC Content on Electro-Optical Properties of PDLC

**Figure 3:**
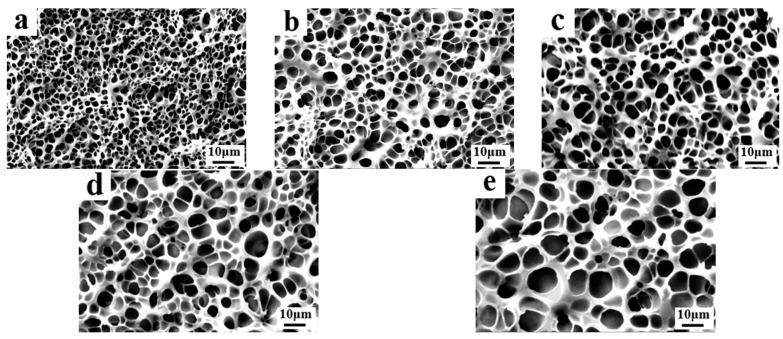
The microscopic mesh distribution of polymer matrix in samples B1–B5: (**a**) 45% SLC-1717; (**b**) 50% SLC-1717; (**c**) 55% SLC-1717; (**d**) 60% SLC-1717; (**e**) 65% SLC-1717.

As one of the critical components during the formation process of PDLC films, the content of LC has a remarkable influence on the micro-morphology of the polymer network as well as the electro-optical properties of PDLC. Generally, the variation in LC content plays a crucial role concerning the modulation of morphological mesh distribution of LC microdroplets within a polymer matrix. The compositions of the samples used for the study are given in Table 1, B1–B5. Figure 3 shows the SEM images of the micro-distribution of the polymer meshes in PDLC with different LC contents. It can be seen from the SEM images that the mesh size gradually increased as the content of LC in the system increased from 45% to 65%. This phenomenon can be attributed to the diluting effect of LCs on the acrylic monomers and the variation in LC content significantly affected the rate of the polymerization of monomers in the system. As the LC content increased from 45% to 65%, the total amount of monomers in the system declined, which caused the photo-induced polymerization of monomers to become slow, resulting in the formation of large LC droplets during phase separation and thus enlarging the pore sizes.

**Figure 4:**
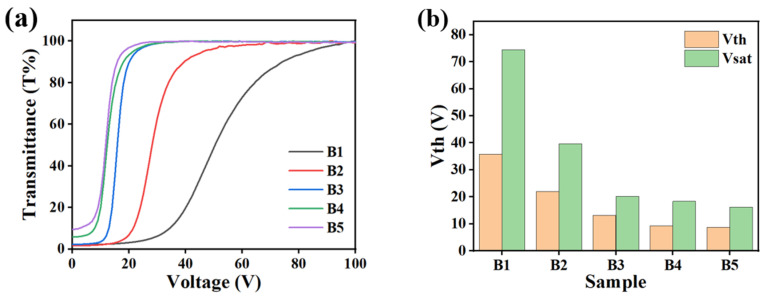

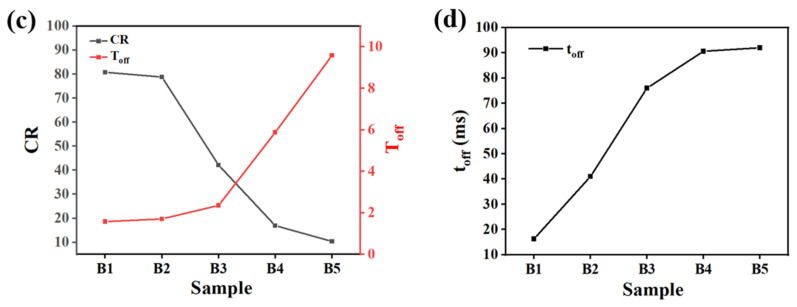
The effect of different LC content on electro-optical properties. (**a**) V–T curve; (**b**) V_th_ and V_sat_; (**c**) CR and T_off_; (**d**) t_off_.

Figure 4 presents the effect of different LC contents on the electro-optical properties of PDLC films. Figure 4a shows the V–T curves for each sample in group B. Obviously, as the LC content increased, the V–T curve progressively shifted to the upper left, but the PDLCs were all still able to realize the transition from an opaque state to a transparent state. Figure 4b shows the corresponding gradual decrease in V_th_ and V_sat_ as the LC content increased from 45% to 65%. Based on the analysis of the above SEM image results, the pore size of the polymer network gradually increased with the increase in the LC content. As a result, the number of interfaces between the LC molecules and the polymer’s matrix were reduced, leading to a reduction in the surface anchoring force exerted by the polymer network on the LC microdroplets. Thus, the driving voltage required to achieve the re-orientation of the LC molecules decreased gradually. Figure 4c demonstrates that as the LC content increased, T_off_ increased, while the CR decreased. The reason for the phenomenon was that the increase in the pore size of the polymer network diminished the light scattering occurring at the interfaces using the polymer matrix and LC molecules. When the samples were illuminated with incident light, the T_off_ increased and the CR decreased due to the reduction in scattered light. Figure 4d indicates that the t_off_ of the samples in group B was gradually prolonged. This can be attributed to the fact that the anchoring force of the polymer network to the LC molecules decreased with the growth of the LC microdroplet size. When the applied voltage was removed, the pointing vectors of the LC molecules did not easily return to their initial state, leading to an increase in the t_off_ [54].

### 2.3. The Effect of Fluorescent Dye Content on Electro-Optical Properties of PDLC

**Figure 5:**
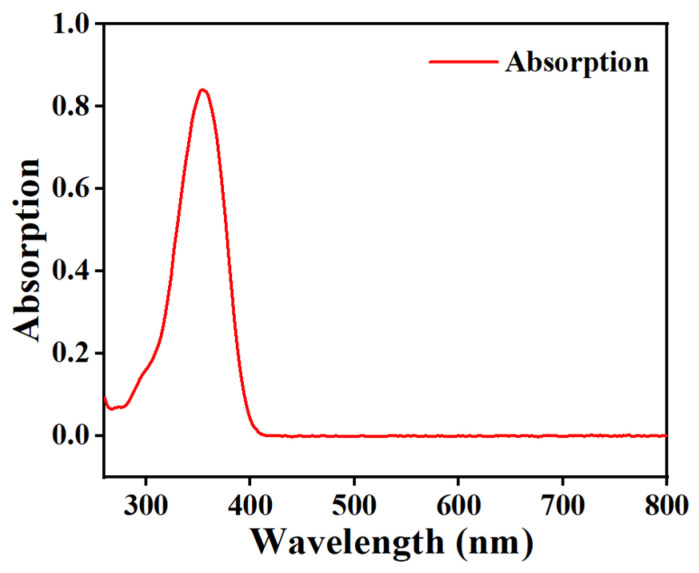
Absorption peak of fluorescent dye AMCA (solvent: isopropanol, concentration: 10^−5^ mol/L).

**Figure 6:**
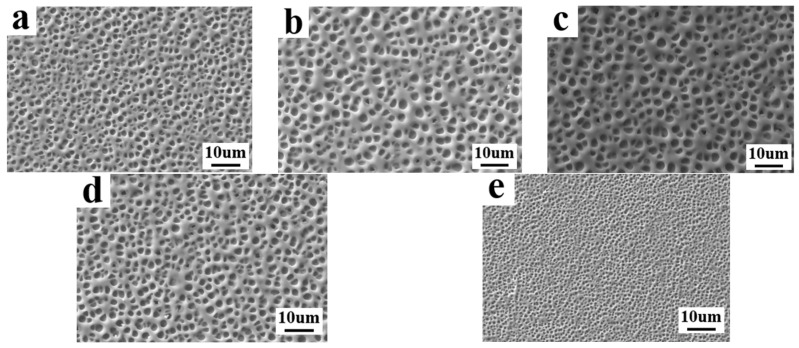
The microscopic mesh distribution of polymer matrix in samples C1–C5: (**a**) 0.1% AMCA; (**b**) 0.2% AMCA; (**c**) 0.3% AMCA; (**d**) 0.4% AMCA; (**e**) 0.5% AMCA.

In this section, the 7-amino-4-methylcoumarin (AMCA), possessing fluorescent properties, was added into the PDLC components, and the effects of different content levels of AMCA on the electro-optical properties as well as the microstructure of PDLC were systematically investigated. The composition of the samples used for the study is given in Table 1, C1–C5. The absorption spectrum of AMCA is shown in Figure 5, which has a distinct absorption peak located in the ultraviolet (UV) region and possesses the capability of emitting blue fluorescence under UV irradiation. Figure 6a–e illustrate SEM images of the mesh of a polymer network containing 0.1%, 0.2%, 0.3%, 0.4%, and 0.5% AMCA, respectively. From the figure, it is obvious that the dimension of the mesh increased and then decreased with the increase in AMCA content and realized its maximum value at 0.2%. 

**Figure 7:**
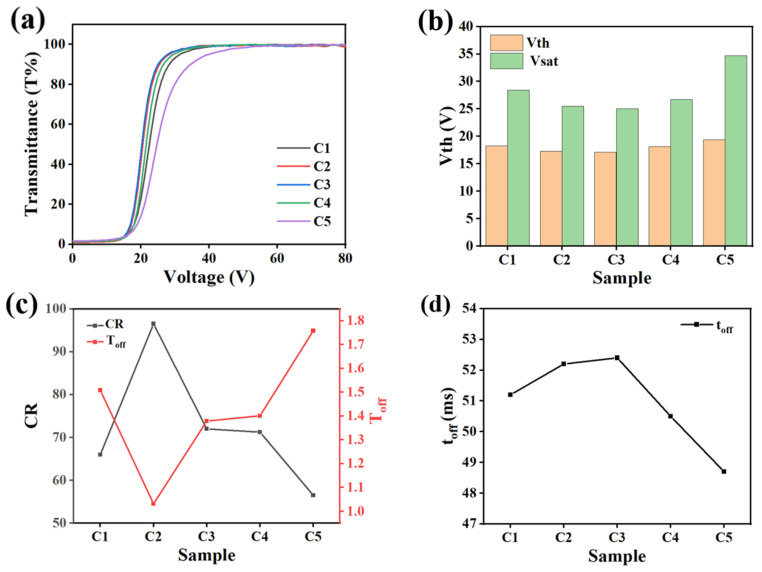
The effect of different AMCA contents on electro-optical properties. (**a**) V–T curve; (**b**) V_th_ and V_sat_; (**c**) CR and T_off_; (**d**) t_off_.

Figure 7 shows the variation of the electro-optical properties of C1–C5 with an increase in AMCA content in the PDLC system. From the V–T curves in Figure 7a, it is evident that each curve with a basic change trend of PDLC is still available, and all can realize the electro-control transition between the scattered state and the transparent state, which indicated that the introduction of AMCA had not altered the basic electro-optical performance of the PDLC films. Figure 7b demonstrates the variation trend of V_th_ and V_sat_ for Group C samples. Both V_th_ and V_sat_ showed a decrease followed by an increase with the increasing AMCA content. When the content of AMCA increased from 0.1% to 0.2%, both the V_th_ and the V_sat_ gradually decreased. This was attributed to the fact that the progressively increasing size of the polymer pores caused a reduction in the amount of interfaces between the polymer matrix and the LC molecules. Therefore, the anchoring effect of the polymer network subjected on the LC molecules was reduced, resulting in a decrease in the voltage required to complete the orientation deflection to the initial state. As the AMCA content continued to increase to 0.5%, the V_th_ and the V_sat_ gradually increased. The reason for this was the gradual decrease in the dimensions of the polymer pores, which led to an increase in the amount of interfaces between the polymer matrix and the LC molecules, resulting in an increase in the driving voltage required for the LC molecules to return to their initial state. Figure 7c demonstrates that the T_off_ of the PDLC film decreased and then increased with the increase in AMCA, whereas the CR changed in the opposite way. The T_off_ value of PDLC films primarily depended on the morphology of the polymer network and the level of refractive index matching. The introduction of AMCA into the PDLC compound system increased the amount of interfaces between the polymer matrix and the LC microdroplets, leading to a stronger ability of the PDLC film to scatter incident light, which resulted in a lower T_off_ and a higher CR [55]. However, as the content of AMCA gradually increased within the system, the corresponding pore size of the polymer network gradually increased. As a result, the scattering effect of incident light by the PDLC system was weakened, which led to an increase in T_off_ and a decrease in CR. Figure 7d exhibits the changing trend of t_off_ at different AMCA contents. As the AMCA content increased, t_off_ first increased and then decreased. This was due to the weaker anchoring effect of the polymer network with large pore sizes on the LC molecules, resulting in a lower driving voltage required to restore the LC microdroplets to their initial state using molecular deflection. The opposite result occurred when the network pore size was small.

**Figure 8:**
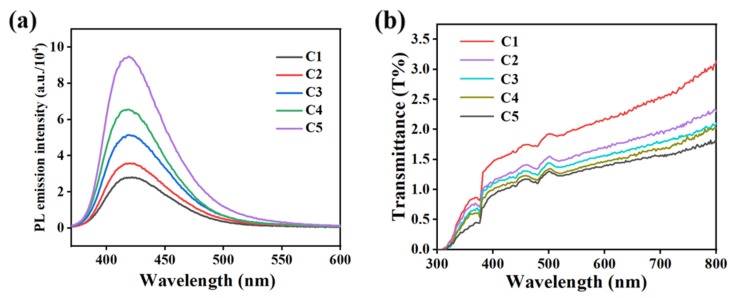
(**a**) Fluorescence emission spectra and (**b**) off-state transmittance curves at different AMCA contents.

As shown in Figure 8a, the fluorescence emission intensity of C1–C5 samples exhibited an obvious upward trend with the increase in AMCA in the system, and the fluorescence emission peak appeared near 420 nm. It can be attributed to the fact that the presence of AMCA enabled the samples to have a fluorescence emission capability near 420 nm, and the intensity increased along with the increase in AMCA content. Figure 8b shows the transmission spectra of different contents of AMCA at 300–800 nm in the off-state. The gradual decrease in transmittance was a result of the gradual mismatch of refractive indices within the system due to the introduction of AMCA.

### 2.4. The Effect of UV-Light Intensity on Electro-Optical Properties of PDLC

**Figure 9:**
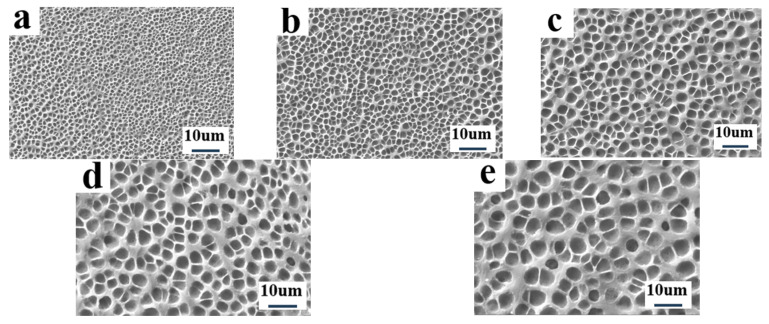
The microscopic mesh distribution of polymer matrix in samples D1–D5: (**a**) 1mW/cm^2^; (**b**) 5mW/cm^2^; (**c**) 10mW/cm^2^; (**d**) 15mW/cm^2^; (**e**) 20mW/cm^2^.

The UV-light intensity significantly affected the electro-optical properties of PDLC films. In this part, PDLC samples D1–D5 were prepared by changing UV-light intensities to explore the effect of different UV-light intensities on the electro-optical properties of PDLC films. Figure 9 shows the SEM images of the micromorphology of the PDLC films obtained using polymerization under different intensities of UV light. It can be observed that the pore size of the polymer network gradually increased from Figure 9a to Figure 9e. Meanwhile, the increase in the network pore size also caused the distribution of the pore size to be non-uniform to some extent. This was due to the fact that the polymerization process slowed down under low light intensity, which allowed the monomers to undergo sufficient polymerization to form a dense network of small pores. However, as the light intensity increased, the polymerization rate of the monomers gradually accelerated, and the polymerization was completed before the monomers had time to diffuse and cross-link fully, resulting in a gradual increase in the mesh size and a certain degree of non-uniformity in its distribution.

**Figure 10:**
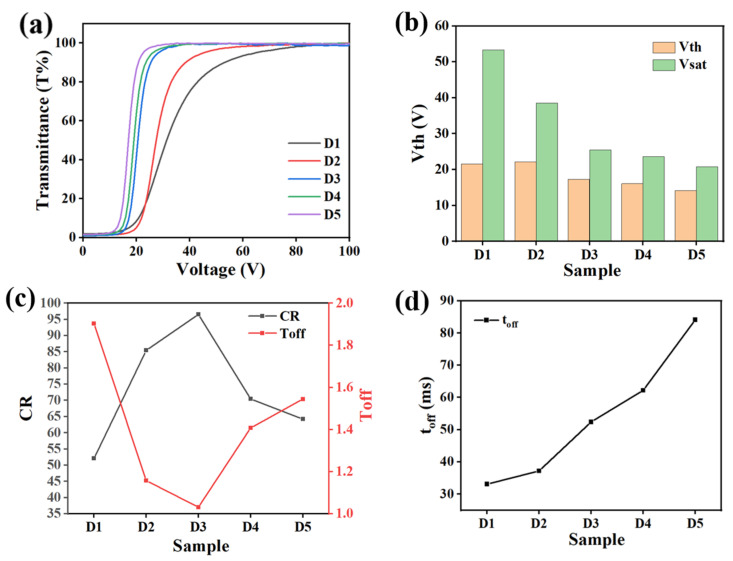
The effect of UV-light intensity on electro-optical properties. (**a**) V–T curve; (**b**) V_th_ and V_sat_; (**c**) CR and T_off_; (**d**) t_off_.

Figure 10 shows that significant electro-optical performance discrepancies in PDLC films resulted from different polymerization-light intensities. Figure 10a presents the electro-optical characteristic curves of Group D samples. From the curve distribution as well as the trend, it can be seen that the difference in electro-optical properties caused by light intensity was more obvious. With the increase in light intensity, the curve gradually shifted to the left, but it was still able to realize the electro-optical conversion between opaque state and transparent state. Figure 10b reveals that the driven voltages V_th_ and V_sat_ gradually decreased from D1 to D5. This was due to the fact that the enlargement of the microscopic polymer pores weakened the anchoring force of the polymer network to the LC molecules, resulting in a decrease in the driving voltage required to complete the deflection to achieve the transparent state by electrifying the LC molecules. Figure 10c illustrates the variation curves of T_off_ and CR for the samples of group D at different polymerization-light intensities. It is not difficult to see that T_off_ decreased and then increased with the increase in light intensity, and the change of CR produced the opposite effect. This was probably due to the difference in the refractive index matching of the system induced at different light intensities, with the system mismatch being greatest at 10 mW/cm^2^, resulting in the lowest T_off_ and the highest CR. Due to the gradual decrease in the number of polymer pores, the force on the LC molecules gradually decreased. Therefore, the time taken for the LC molecules to return to their initial state increased when the electric field was withdrawn, as shown in Figure 10d.

**Figure 11:**
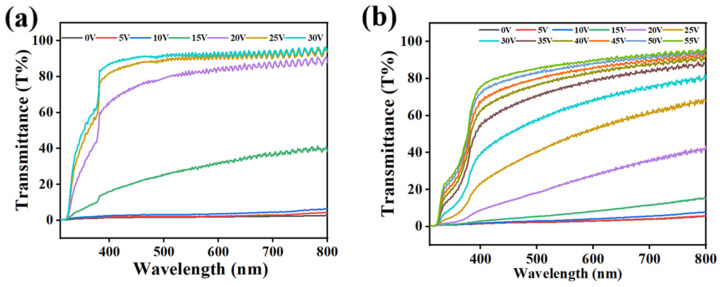

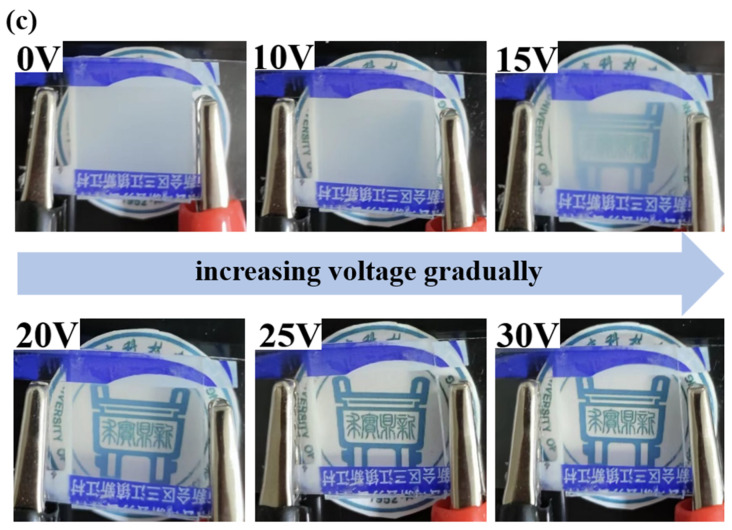
(**a**) Transmittance curves of sample C2 at different voltages. (**b**) Transmittance curves of sample D1 at different voltages. (**c**) Stepwise-driven display of C2.

In its energized state, the progressive drive display of the PDLC can be controlled with the magnitude of the applied voltage. Figure 11 presents the variation in transmittance of C2 and D1 samples at different voltages. The variation in the transmittance of sample C2 at different voltages is shown in Figure 11a, where C2 was progressively driven from a fully scattered state to a transparent state when the applied voltage was increased from 0 V to 30 V. Similarly, when the voltage was increased from 0 V to 55 V, the electro-controlled variation of transmittance was also realized for D2, as shown in Figure 11b. Figure 11c displays the transmittance picture of sample C2 at different voltages. C2 gradually become transparent with the increase in the applied voltage, which is in accordance with the curve variation of Figure 11a. The above results collectively indicate that using the difference in driving voltage of samples can result in different transmittance levels, laying the foundation for intelligent stepwise electronic control. 

### 2.5. The Effect of Partitioned Polymerization on Electro-Optical Properties of PDLC

**Figure 12:**
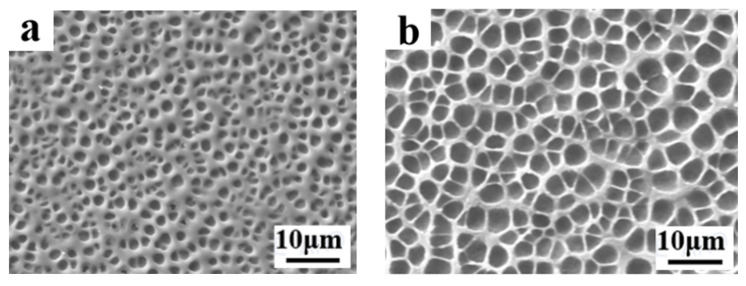
The microscopic mesh distribution of polymer matrix under different light intensity: (**a**) 1 mW/cm^2^; (**b**) 20 mW/cm^2^.

**Figure 13:**
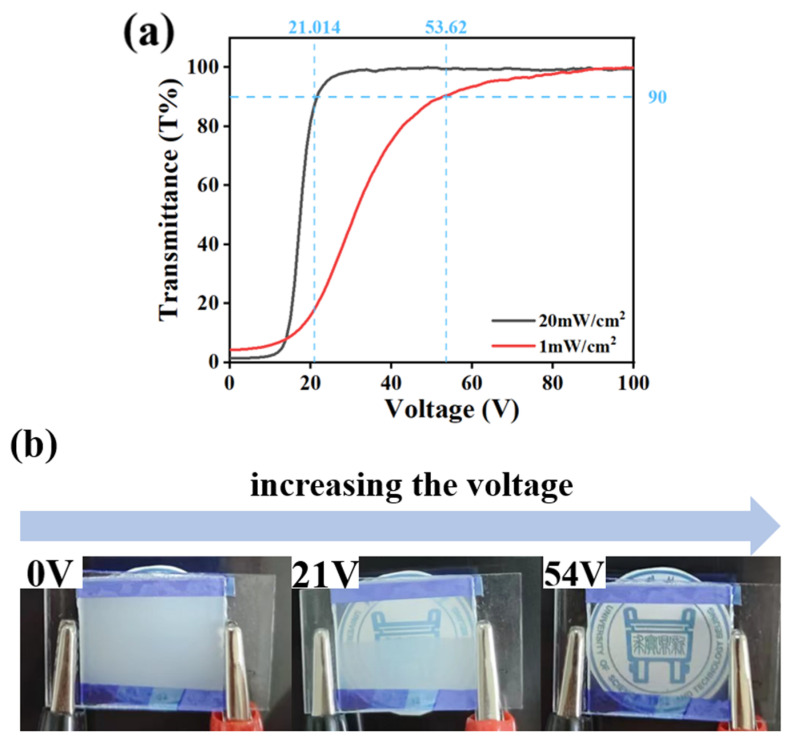
(**a**) Measured V–T curves at 1 mW/cm^2^ and 20 mW/cm^2^; (**b**) partitioned stepwise display of PDLC device.

Modulation of the electro-optical performance of different regions of a single-PDLC device by differentiating each regional component tended to require the implementation of advanced technological support. More satisfactory results probably can be obtained by altering the external conditions rather than by changing the compositions of the internal components of the system. Inspired by the effects of UV-light intensity on the electro-optical properties of PDLC discussed above, different regions of the sample were polymerized using different UV-light intensities, thereby causing differences in the driving voltage in achieving a partitioned stepwise-driven display under different voltages. Figure 12a,b show the microstructure of a polymer network formed using polymerization in two different regions of PDLC at light intensities of 1 mW/cm^2^ and 20 mW/cm^2^, respectively. From Figure 12, it can be noticed that the mesh in the region polymerized using low light intensity was small and dense, while the mesh in the region polymerized using high light intensity was large and sparse. The reason for this phenomenon was the same as mentioned before. In order to study the electro-optical properties of these two regions at different polymerization-light intensities, their voltage–transmittance curves (V–T curves) were measured. In Figure 13a, the red line represents the V–T curve at the light intensity of 1 mW/cm^2^, whereas the black line presents light intensity at 20 mW/cm^2^. It was clear that the V_sat_ of the region polymerized using 1 mW/cm^2^ light intensity was lower at 21.0 V, whereas the voltage of the region polymerized at 20 mW/cm^2^ light intensity was 53.6 V. This result indicated that the two parts of the PDLC device could be driven progressively when a voltage was applied to them. When the voltage was gradually increased to 21.0 V, the transmittance of the region polymerized under high light intensity was the first to reach 90%, whereas the transmittance of the low-light-intensity region was about 18% at this time. Therefore, the region polymerized at high light intensity was the first to be driven to become transparent, while the low-light-intensity region still maintained a high scattering state. This was the first stage of stepwise driving. In the second stage, as the applied voltage was further increased to 53.6 V, the transmittance of the other region polymerized under low light intensity also gradually reached 90%, and the whole PDLC device was driven to transparency, as shown in Figure 13b. This novel approach to driving PDLC devices will facilitate an effortless and convenient method for advanced displays in the future.

## 3. Mechanism and Applications

**Figure 14:**
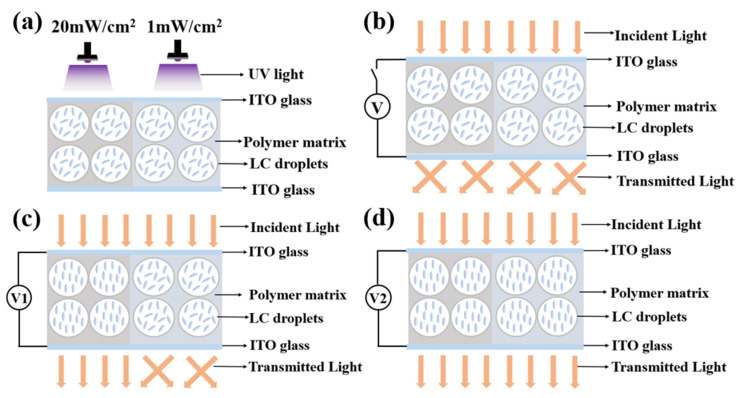
The schematic diagram of partitioned stepwise display of PDLC device (V2 > V1): (**a**) partitioned polymerization; (**b**) no voltage applied, V = 0; (**c**) V1 voltage applied, V1 = 21V; (**d**) V2 voltage applied, V2 = 54V.

The operating principle of the proposed PDLC device is presented in Figure 14. Figure 14a displays the approach to realize partitioned polymerisation, where two regions of the PDLC film are polymerised using two different light intensities. The right region was polymerized at low light intensities and the left region was the opposite. In the voltage-off state, due to the mismatch of the refractive indexes between the LC molecules and the polymer matrix, the incident light was scattered by both regions of the as-polymerized PDLC film, as shown in Figure 14b. The proposed device, in other words, exhibited plenary scattering. When applying a low-voltage V1 between the two ITO electrodes, a homogeneous electric field was applied to the PDLC film. The PDLC film in the region polymerized at high light intensity became transparent, while the PDLC in the region polymerized at low light intensity had a higher V_sat_ due to its smaller pore size and still remained in the scattering state, resulting in a patterned transparent mode, as shown in Figure 14c. As the applied voltage continued to increase from V1 to V2, which was greater than the V_sat_ of PDLC polymerized at low light intensity, all LC molecules in the PDLC films were aligned parallel to the applied electric field, resulting in all the incident light transitioning through without scattering, as well as making the total-transparent state obtained, as presented in Figure 14d. 

**Figure 15:**
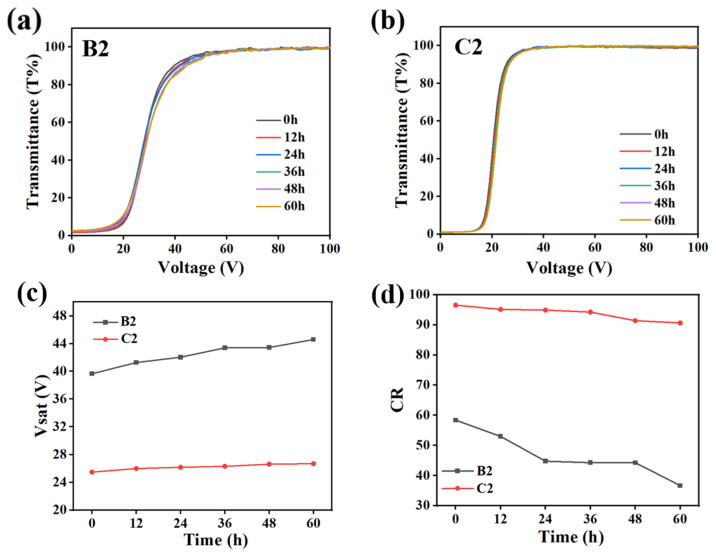
Anti-aging test of samples. (**a**) V–T curves of sample B2; (**b**) V–T curves of sample C2; (**c**) V_sat_ of sample B2 and C2; (**d**) CR of sample B2 and C2.

In order to improve product reliability, anti-aging tests were conducted on samples B2 and C2 in this section. The aging conditions in this test were 25 °C and 0.18 w/m^2^ UV-light intensity. The electro-optical properties of the samples were measured at intervals of 12 h and the experimental results are shown in Figure 15. Figure 15a,b show the V–T curves recorded for samples B2 and C2 after ageing at different durations, respectively. Sample B2 differed from sample C2 due to the addition of AMCA to the latter. From the figure, it can be seen that the electro-optical properties of B2 appeared to be slightly deteriorated at 60 h with the extension of the aging time, while the electro-optical properties of sample C2 were basically unchanged. The variations of the V_sat_ and CR of B2 and C2 are demonstrated under different durations in Figure 15c,d, respectively. Noticeably, the C2 sample exhibited more minute fluctuations with aging time compared to sample B2 without AMCA, which suggested that the presence of AMCA conferred samples with better environmental tolerance. The reason for this phenomenon was that AMCA has a certain UV-absorption capacity, which can absorb part of the UV light and thus slowed down the aging damage of PDLC samples.

**Figure 16:**
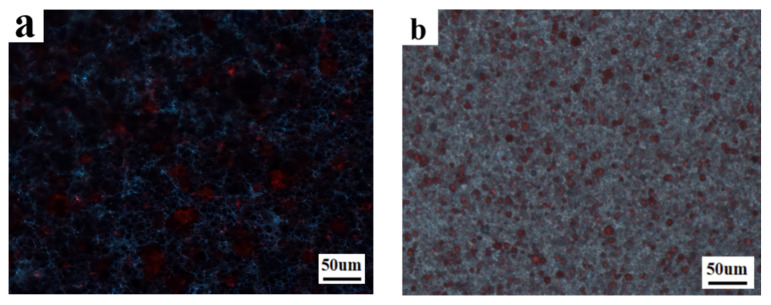

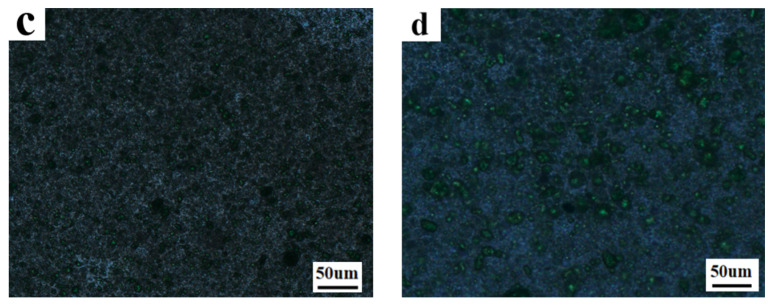
POM images of thermochromic materials at different temperatures. (**a**) Red thermochromic materials at 20 °C. (**b**) Red thermochromic materials at 60 °C. (**c**) Green thermochromic materials at 20 °C. (**d**) Green thermochromic materials at 60 °C.

**Figure 17:**
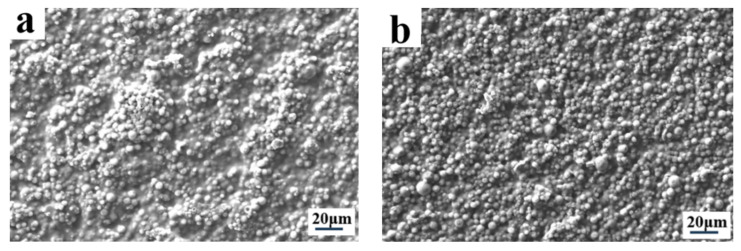
SEM images of thermochromic materials. (**a**) Red thermochromic materials. (**b**) Green thermochromic materials.

Thermochromic materials play an instrumental role in implementing dynamic variations in color, which refers to a functional material capable of changing its visible-light-absorption spectrum when exposed to hot or cold conditions, with the property that color varies with temperature. The temperature at which a color change occurs was called the thermochromic temperature (T_0_). In this section, two thermochromic materials, R and G, with a T_0_ of 45 °C, were selected. When the temperature was below 45 °C, R and G are curry color and brown color, respectively. As temperature rose above 45 °C, the former changed to red and the latter to green. Figure 16 records the coloration behavior of two thermochromic materials using POM at different temperatures. Figure 16a,c visually presents that when the temperature was at room temperature, 20 °C or below T_0_, R and G showed curry and brown, respectively. Then, the former turned to red and the latter to green upon a gradual increase in temperature to 60 °C above T_0_. Figure 17 exhibits the SEM images of the two thermochromic materials, from which it can be seen that the morphology presented a rounded spherical shape, with particle sizes ranging from 1 to 8 micrometers and with a relatively uniform distribution.

**Figure 18:**
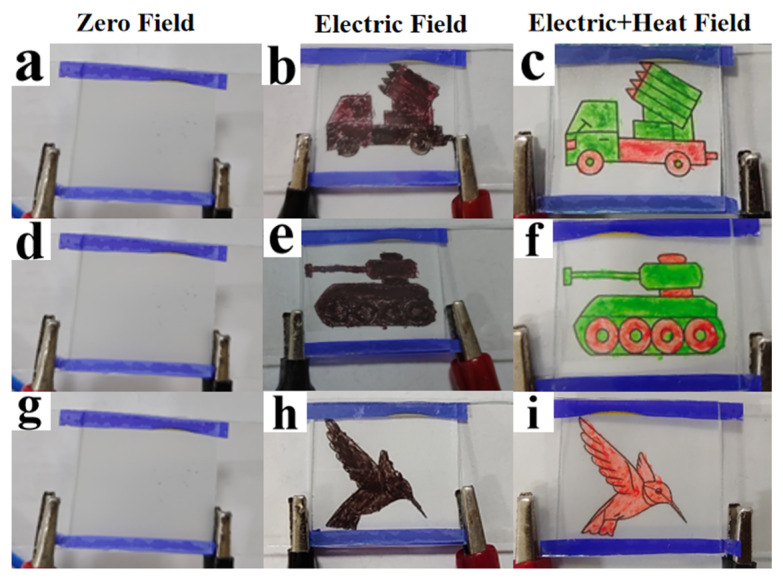
Optical display behavior of sample C3 under different fields, (**a**) zero field (0 V, 20 °C); (**b**) electric field (30 V, 20 °C); (**c**) electric + heat field (30 V, 60 °C); (**d**) zero field (0 V, 20 °C); (**e**) electric field (30 V, 20 °C); (**f**) electric + heat field (30 V, 60 °C); (**g**) zero field (0 V, 20 °C); (**h**) electric field (30 V, 20 °C); and (**i**) electric + heat field (30 V, 60 °C).

**Figure 19:**
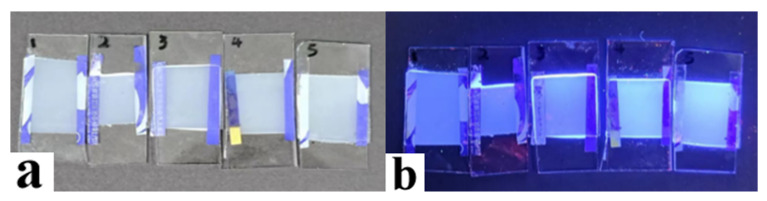
Fluorescence display from sample C1 to sample C5: (**a**) sunlight exposure; (**b**) UV-light exposure.

Using the above two thermochromic materials as patterned backing substrates, optical display behaviors under different fields were investigated, as shown in Figure 18. At the zero field, as illustrated in Figure 18a,d,g, the PDLC device showed a scattering state, and the backside pattern behind it was not shown. When an electric field higher than V_sat_ was applied, the PDLC device changed from a scattering state to a transparent state, and an image patterned using a thermochromic material behind it appeared. By simultaneously applying a thermal field higher than T_0_ in the presence of the electric field described above, the backside pattern underwent electrochromic variation and rich pattern variation was realized. Figure 19 demonstrates the fluorescence display of PDLC films containing different levels of AMCA under UV irradiation, and the fluorescence brightness became stronger and stronger with the increase in AMCA content, which was in correspondence with the experimental results mentioned above. 

**Figure 20:**
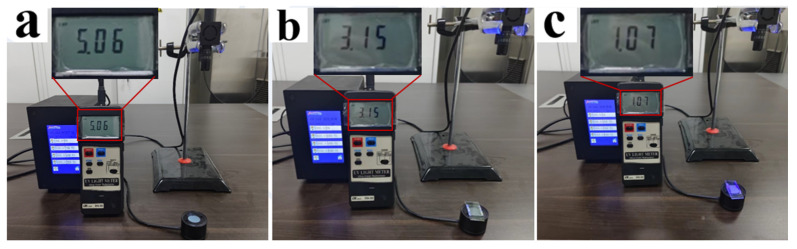
UV-shielding test: (**a**) blank sample; (**b**) B2 sample; and (**c**) C2 sample.

**Figure 21:**
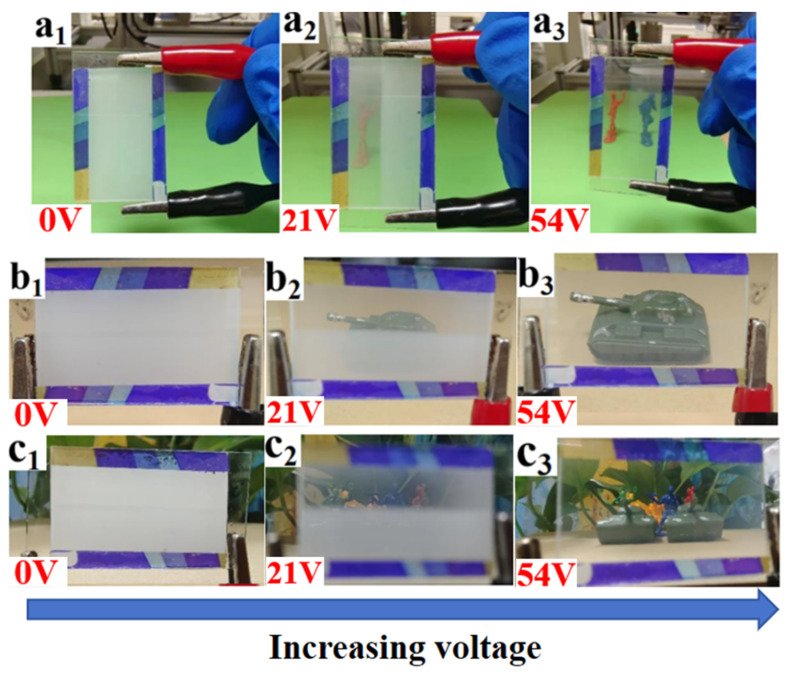
Demonstration of an electronically controlled partition-driven model: (**a_1_**) apply 0V voltage; (**a_2_**) apply 21V voltage; (**a_3_**) apply 54V voltage; (**b_1_**) apply 0V voltage; (**b_2_**) apply 21V voltage; (**b_3_**) apply 54V voltage; (**c_1_**) apply 0V voltage; (**c_2_**) apply 21V voltage; (**c_3_**) apply 54V voltage.

Figure 5 demonstrates that AMCA has a distinguishable UV-absorption peak, thus the PDLC films containing AMCA can be used to absorb part of the incident UV light by using the absorption of AMCA during UV-light irradiation, providing UV-shielding properties to PDLC films. To verify this conjecture, the UV-control shielding experiments were carried out using a UV lamp with a UV-intensity measurement as shown in Figure 20. The incident UV-light intensity was kept constant in the experiment by keeping the distance from the UV-emitting-light source to the UV sensor unchanged. When no sample was placed on the UV sensor as a control in Figure 20a, the UV sensor measured the UV intensity emitted from the light source to be 5.06 mW/cm^2^. When sample B2 without AMCA was placed on the UV sensor as shown in Figure 20b, the UV intensity emitted from the light source measured using the UV sensor was 3.15 mW/cm^2^. And, when sample C2 containing AMCA was placed on the UV sensor as shown in Figure 20c, the UV intensity was 1.07 mW/cm^2^. The UV-shielding efficiencies of samples B2 and C2 were 37.75% and 78.85%. The only difference between samples B2 and C2 was that the latter contained AMCA, and the results showed that the addition of AMCA increased the UV-shielding efficiency of the PDLC films by 41.1%. This result is attributed to the fact that the AMCA in the PDLC film reduced the amount of light transmitted through the sample by absorbing some incident UV light, thus achieving a certain degree of shielding from external UV light. It also better explained that the AMCA-containing sample C2 showed better anti-aging performance under the above UV-aging-test condition. Figure 21 demonstrates a partitioned electrically controlled display utilizing PDLC films. As the voltage was gradually increased, different regions of the PDLC film were driven successively, and the subsequent images were revealed. These applications exemplified the potential of this new PDLC film for advanced displays.

## 4. Experiment

### 4.1. Materials

**Figure 22:**
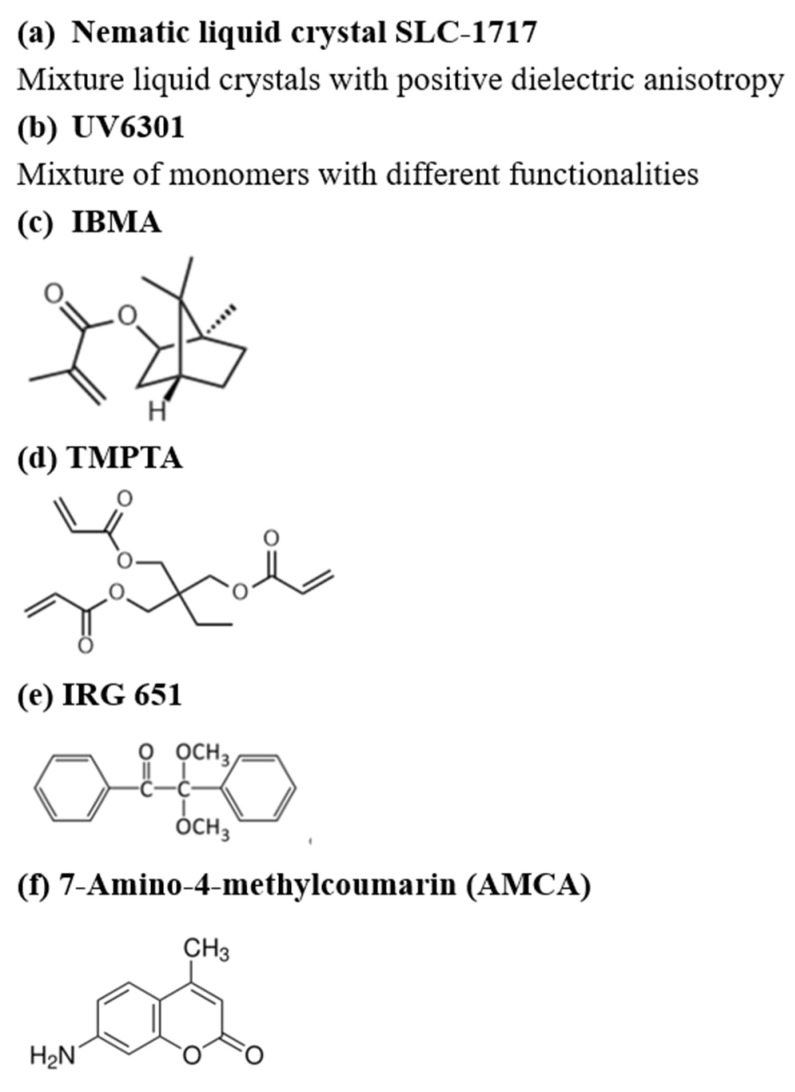
The molecular structures of the mentioned experimental materials.

We used the following materials: a commercial nematic liquid crystal (SLC-1717, n_e_ = 1.720, n_o_ = 1.519, Δn = (n_e_ − n_o_) = 0.201, Shijiazhuang Chengzhi Yonghua Display Material Co., Ltd., Shijiazhuang, China); a polymer matrix (UV6301, Han Rui Industrial Co., Ltd., Shanghai., China); a monofunctional monomer isobornyl methacrylate (IBMA, Anhui Zesheng Technology Co., Ltd., Anqing, China); a trifunctional monomer trimethylolpropane triacrylate (TMPTA, Anhui Zesheng Technology Co., Ltd., Anqing, China). UV-6301, IBMA, and TMPTA were all used as cross-linkers in this experiment. The fluorescent material was 7-Amino-4-methylcoumarin (AMCA, Anhui Zesheng Technology Co., Ltd., Anqing, China). We also used the following materials: a thermochromic material (Shenzhen Oriental Colour Change Co., Ltd., Shenzhen, China); a free-radical photoinitiator (IRG651, Anhui Zesheng Technology Co., Ltd.). The molecular structures of the above materials are shown in Figure 22 below.

### 4.2. Sample Preparation

In the experiment, the polymerization-induced phase separation (PIPS) technology and partitioned preparation technology were both employed in preparing the step-driven display PDLC film. The detailed process is as follows: 

(a) First, SLC-1717/UV-6301/IBMA/TMPTA/IRG651 was precisely weighed in a 2 mL centrifuge tube. Next, the mixed solution was pre-shocked on a rotary oscillator for 3 min at room temperature and subsequently sonicated at 60 °C for 20 min. This process should be carried out several times until a homogeneous mixture solution is obtained. Similarly, in order to prepare the PDLC film containing AMCA, SLC-1717/UV-6301/IBMA/TMPTA/IRG651/AMCA was precisely weighed in a 2 mL centrifuge tube at first. Next, the mixed solution was pre-shocked on a rotary oscillator for 5 min at room temperature and subsequently sonicated at 60 °C for 30 min. This process should be performed several times until the homogeneous SLC-1717/UV-6301/IBMA/TMPTA/IRG651/AMCA mixture solution is obtained.

(b) We took two pieces of 2 cm × 3 cm ITO glass and measured the conductive surfaces of them with an ammeter. ITO glass surfaces should be clean and free of impurities after washing. We put the conductive surfaces of the two pieces of ITO glass opposite each other, separated by a polyethylene terephthalate film of about 20 um thickness on both sides. The strong glue 502 was utilized to bond the two sides of the ITO glasses to finish the fabrication of the LC cell. Then, the above homogeneous SLC-1717/UV-6301/IBMA/TMPTA/IRG651 mixture solution was filled slowly into the as-prepared LC cell using capillary siphoning. Similarly, for the preparation of the PDLC film containing AMCA, the above homogeneous SLC-1717/UV-6301/IBMA/TMPTA/IRG651/AMCA mixture solution was filled slowly into the as-prepared LC cell using capillary siphoning.

(c) Finally, one-step-driven PDLC films with fluorescent capability were obtained through irradiation with a commercial 365 nm UV curing lamp at 10 mW/cm^2^ for 10 min. Specifically, step-driven PDLC film was prepared by curing two regions of the LC cell for 10 min using different UV-light intensities. The UV-curing-light intensities used for the two regions in this experiment were 1 mW/cm^2^ and 10 mW/cm^2^. Similarly, for the curing of the PDLC film containing AMCA, the process is the same. Notably, the entire polymerization process was performed at room temperature, and the amount of IRG651 content was the same as the percentage of the total amount of the mixed solution SLC-1717/UV-6301/IBMA/TMPTA. The sample composition is shown in Table 1.

### 4.3. Measurement

In order to observe the micro-distribution of the polymer network of the PDLC samples, the samples were first cut and processed into 0.5 cm × 0.5 cm pieces and then eluted from the LC molecules by soaking them in an enclosed glass vessel filled with cyclohexane for about 14 days. The soaked samples were dried naturally and then sprayed with gold for 70 s for observation in the SEM (ZEISS SUPRA55). The comprehensive LC parameter tester (LCT-5016) was used to characterize the electro-optical properties of each PDLC sample. Electro-optical properties are critical parameters that describe the behavior of PDLC in practice, including the saturation voltage (V_sat_), threshold voltage (V_th_), off-state transmittance (T_off_), contrast ratio (CR), and response time (t_off_). CR is defined as the ratio of the on-state (T_on_) transmittance to the off-state transmittance (T_off_) of the PDLC: T_on_/T_off_, where T_off_ is the transmittance when the PDLC is completely switched off without applying the electric field and T_on_ is the transmittance when the PDLC is completely switched on after applying enough of the electric field. The UV-Vis-NIR spectrometer (JASCO V-570) was utilized to characterize the absorption spectra of fluorescent dye AMCA and the transmission spectra of the PDLC samples. The fluorescence emission spectra of the PDLC samples were detected using the transient-steady-state fluorescence spectrometer (FLS 1000). The anti-aging properties of the samples of the PDLC samples were conducted in the UV-accelerated-aging tester.

## 5. Conclusions

In conclusion, a novel step-driven PDLC transparent display with fluorescent capacity using partitioned polymerization was proposed in this paper. Acting as an excellent fluorescent material, AMCA provided PDLC with outstanding electro-optical behavior while reducing V_sat_ by 14.2 V and increasing CR by 38.2, realizing a 35.8% decrease in V_sat_ and 65.4% improvement in CR. Meanwhile, it was found that the change of polymer pore size caused by varying components and experimental conditions had a remarkable influence on the electro-optical performance of PDLC film. Thereafter, inspired by the fact that light intensity enabled differentiation in the driving voltage, by exposing the different regions of the LC cell to different UV-light intensities, significant differentiation of the microscopic pore sizes in the corresponding regions of the PDLC was achieved, thus enabling modulation in the three states of total scattering, semi-transparent, and total transparent at appropriate voltages. In addition, the underlay of the transparent display was mapped using thermochromic materials, enabling the PDLC to demonstrate variable patterns in the presence of multiple external field superimpositions. Notably, the presence of AMCA endowed PDLC film with outstanding UV shielding of up to 78.8% and brilliant aging stability in the 60 h test. Therefore, this study provided a methodological reference to break the current single-PDLC driving model.

## Figures and Tables

**Table 1:** Compositional formulation of each PDLC sample.

	SLC-1717 (%)	UV-6301 (%)	IBMA (%)	TMPTA (%)	AMCA (%)	IRG651 (%)	UV-Light Intensity (mW/cm^2^)
A1	50	49	0	1	0	0.5	10
A2	50	48	1	1	0	0.5	10
A3	50	47	2	1	0	0.5	10
A4	50	46	3	1	0	0.5	10
A5	50	45	4	1	0	0.5	10
B1	45	55 ^a^			0	0.5	10
B2	50	50 ^a^			0	0.5	10
B3	55	45 ^a^			0	0.5	10
B4	60	40 ^a^			0	0.5	10
B5	65	35 ^a^			0	0.5	10
C1	50	50 ^a^			0.1	0.5	10
C2	50	50 ^a^			0.2	0.5	10
C3	50	50 ^a^			0.3	0.5	10
C4	50	50 ^a^			0.4	0.5	10
C5	50	50 ^a^			0.5	0.5	10
D1	50	50 ^a^			0.2	0.5	1
D2	50	50 ^a^			0.2	0.5	5
D3	50	50 ^a^			0.2	0.5	10
D4	50	50 ^a^			0.2	0.5	15
D5	50	50 ^a^			0.2	0.5	20

^a^ UV-6301:IBMA:TMPTA = 48:1:1. (For a given total number of monomers, where the ratio of different monomers is UV-6301:IBMA:TMPTA = 48:1:1. For example, 50 a represents a case where the total amount of monomers in the system is 50%, and the proportion of UV-6301:IBMA:TMPTA in the 50% monomers is 48:1:1).

## Data Availability

Research data are not shared.
